# Supplementary material for: Unveiling the Bioactive Potential of the Invasive Jellyfish Phyllorhiza punctata Through Integrative Transcriptomic and Proteomic Analyses
Source: Biomolecules. 2025 Aug 4;15(8):1121. doi: 10.3390/biom15081121 (PMC12383608; doi:10.3390/biom15081121)
Supplement: Supplementary file 1 [file biomolecules-15-01121-s001.zip › Suplementary Material/FileS3/PEPFOLD4/XP_065059832_7823/clusters.html]

 PEP-FOLD clusters


Download archive of all models  here   

|  |  |  |
| --- | --- | --- |
| # | file | sOPEP |
| # cluster 1 - Size: 89 |  |  |
|  | XP\_065059832\_7823-model1 | -29.3364 |
|  | XP\_065059832\_7823-model1.1 | -29.2206 |
|  | XP\_065059832\_7823-model1.2 | -29.2128 |
|  | XP\_065059832\_7823-model1.3 | -29.1966 |
|  | XP\_065059832\_7823-model1.4 | -29.1803 |
|  | XP\_065059832\_7823-model1.5 | -29.1592 |
|  | XP\_065059832\_7823-model1.6 | -29.1142 |
|  | XP\_065059832\_7823-model1.7 | -29.1006 |
|  | XP\_065059832\_7823-model1.8 | -29.086 |
|  | XP\_065059832\_7823-model1.9 | -29.0779 |
|  | XP\_065059832\_7823-model1.10 | -29.0638 |
|  | XP\_065059832\_7823-model1.11 | -29.0418 |
|  | XP\_065059832\_7823-model1.12 | -29.0099 |
|  | XP\_065059832\_7823-model1.13 | -29.0058 |
|  | XP\_065059832\_7823-model1.14 | -28.9652 |
|  | XP\_065059832\_7823-model1.15 | -28.9217 |
|  | XP\_065059832\_7823-model1.16 | -28.9096 |
|  | XP\_065059832\_7823-model1.17 | -28.8663 |
|  | XP\_065059832\_7823-model1.18 | -28.8504 |
|  | XP\_065059832\_7823-model1.19 | -28.8448 |
|  | XP\_065059832\_7823-model1.20 | -28.8309 |
|  | XP\_065059832\_7823-model1.21 | -28.8298 |
|  | XP\_065059832\_7823-model1.22 | -28.8249 |
|  | XP\_065059832\_7823-model1.23 | -28.8192 |
|  | XP\_065059832\_7823-model1.24 | -28.7464 |
|  | XP\_065059832\_7823-model1.25 | -28.7419 |
|  | XP\_065059832\_7823-model1.26 | -28.7103 |
|  | XP\_065059832\_7823-model1.27 | -28.6894 |
|  | XP\_065059832\_7823-model1.28 | -28.6839 |
|  | XP\_065059832\_7823-model1.29 | -28.6658 |
|  | XP\_065059832\_7823-model1.30 | -28.6447 |
|  | XP\_065059832\_7823-model1.31 | -28.615 |
|  | XP\_065059832\_7823-model1.32 | -28.6012 |
|  | XP\_065059832\_7823-model1.33 | -28.5953 |
|  | XP\_065059832\_7823-model1.34 | -28.5735 |
|  | XP\_065059832\_7823-model1.35 | -28.5562 |
|  | XP\_065059832\_7823-model1.36 | -28.5486 |
|  | XP\_065059832\_7823-model1.37 | -28.5395 |
|  | XP\_065059832\_7823-model1.38 | -28.5051 |
|  | XP\_065059832\_7823-model1.39 | -28.5034 |
|  | XP\_065059832\_7823-model1.40 | -28.4826 |
|  | XP\_065059832\_7823-model1.41 | -28.459 |
|  | XP\_065059832\_7823-model1.42 | -28.4502 |
|  | XP\_065059832\_7823-model1.43 | -28.4339 |
|  | XP\_065059832\_7823-model1.44 | -28.4324 |
|  | XP\_065059832\_7823-model1.45 | -28.3891 |
|  | XP\_065059832\_7823-model1.46 | -28.38 |
|  | XP\_065059832\_7823-model1.47 | -28.362 |
|  | XP\_065059832\_7823-model1.48 | -28.3334 |
|  | XP\_065059832\_7823-model1.49 | -28.3265 |
|  | XP\_065059832\_7823-model1.50 | -28.3216 |
|  | XP\_065059832\_7823-model1.51 | -28.3086 |
|  | XP\_065059832\_7823-model1.52 | -28.2951 |
|  | XP\_065059832\_7823-model1.53 | -28.2823 |
|  | XP\_065059832\_7823-model1.54 | -28.2802 |
|  | XP\_065059832\_7823-model1.55 | -28.2782 |
|  | XP\_065059832\_7823-model1.56 | -28.2517 |
|  | XP\_065059832\_7823-model1.57 | -28.2219 |
|  | XP\_065059832\_7823-model1.58 | -28.2217 |
|  | XP\_065059832\_7823-model1.59 | -28.2106 |
|  | XP\_065059832\_7823-model1.60 | -28.2047 |
|  | XP\_065059832\_7823-model1.61 | -28.195 |
|  | XP\_065059832\_7823-model1.62 | -28.1413 |
|  | XP\_065059832\_7823-model1.63 | -28.1307 |
|  | XP\_065059832\_7823-model1.64 | -28.1216 |
|  | XP\_065059832\_7823-model1.65 | -28.1049 |
|  | XP\_065059832\_7823-model1.66 | -28.0915 |
|  | XP\_065059832\_7823-model1.67 | -28.071 |
|  | XP\_065059832\_7823-model1.68 | -28.0701 |
|  | XP\_065059832\_7823-model1.69 | -28.058 |
|  | XP\_065059832\_7823-model1.70 | -28.0444 |
|  | XP\_065059832\_7823-model1.71 | -27.9557 |
|  | XP\_065059832\_7823-model1.72 | -27.9351 |
|  | XP\_065059832\_7823-model1.73 | -27.9256 |
|  | XP\_065059832\_7823-model1.74 | -27.9156 |
|  | XP\_065059832\_7823-model1.75 | -27.8979 |
|  | XP\_065059832\_7823-model1.76 | -27.8827 |
|  | XP\_065059832\_7823-model1.77 | -27.841 |
|  | XP\_065059832\_7823-model1.78 | -27.8321 |
|  | XP\_065059832\_7823-model1.79 | -27.81 |
|  | XP\_065059832\_7823-model1.80 | -27.8078 |
|  | XP\_065059832\_7823-model1.81 | -27.7596 |
|  | XP\_065059832\_7823-model1.82 | -27.7351 |
|  | XP\_065059832\_7823-model1.83 | -27.6925 |
|  | XP\_065059832\_7823-model1.84 | -27.584 |
|  | XP\_065059832\_7823-model1.85 | -27.4816 |
|  | XP\_065059832\_7823-model1.86 | -27.4278 |
|  | XP\_065059832\_7823-model1.87 | -27.2745 |
|  | XP\_065059832\_7823-model1.88 | -27.1266 |
| # cluster 2 - Size: 9 |  |  |
|  | XP\_065059832\_7823-model2 | -27.5843 |
|  | XP\_065059832\_7823-model2.1 | -27.4216 |
|  | XP\_065059832\_7823-model2.2 | -27.3377 |
|  | XP\_065059832\_7823-model2.3 | -27.3092 |
|  | XP\_065059832\_7823-model2.4 | -27.3059 |
|  | XP\_065059832\_7823-model2.5 | -27.0557 |
|  | XP\_065059832\_7823-model2.6 | -26.9779 |
|  | XP\_065059832\_7823-model2.7 | -26.9569 |
|  | XP\_065059832\_7823-model2.8 | -26.798 |
| # cluster 3 - Size: 1 |  |  |
|  | XP\_065059832\_7823-model3 | -26.2924 |
| # cluster 4 - Size: 1 |  |  |
|  | XP\_065059832\_7823-model4 | -20.4978 |
|  |  |  |
